# Modified New [0, 1] - Right Truncated Exponential Rayleigh Distribution Structure and Application

**DOI:** 10.12688/f1000research.174089.1

**Published:** 2026-01-21

**Authors:** Maysaa J. Mohammed, Ali T. Mohammed, Rehab N. shalan

**Affiliations:** 1 Department of Mathematics, University of Baghdad College of Education for Pure Science Ibn Al-Haitham, Baghdad, Iraq; 2 Department of Mathematics, University of Baghdad, College of Science, Baghdad, Iraq

**Keywords:** Right – truncated [0, 1], exponential Rayleigh distribution, quantile function, Order Statistics, Entropy, Simulation

## Abstract

**Background:**

In general, truncated distributions provide a flexible framework that allows for more realistic and accurate representation of constrained data compared to using non-truncated distributions, making them a fundamental element in modern statistical models and the analysis of constrained data.

**Methods:**

Right – truncated [0,1] exponential Rayleigh distribution ([0,1]-RTERD) for lifetime is presented in this paper. The new distribution contains two parameters, one of scale and another for the shape. Statistical functions of the (RTERD), such as cumulative functions (CDF), probability density (PDF), survival, and hazard functions, are presented in terms of mathematical construction and graphics. Besides, discuss the mathematical and statistical properties of the new distribution, including median, moments of the origin, coefficients of skewness and kurtosis, order statistic, moment generating function, the rėnyi entropy, quantile function.

**Results:**

the parameters of this life expectancy distribution will be estimate using the maximum likelihood estimation method and compare new continuous model with other continuous distributions using statistical information criterion. Finally, apply the ([0,1]-RTERD) to actual datasets to validate the model’s practicality. The ([0,1]-RTERD) frequently produces a better fit and shows more modeling precision when handling lifespan and reliability data, according to comparisons with traditional models.

**Conclusions:**

The [0,1]-RTERD provides a new framework for more accurately dealing with data restricted to the interval [0,1], where it is characterized by several important properties, including changing the shape, center, and width of the original distribution as a result of the normalization process, which is reflected in the expected value, standard deviation, and other instantaneous properties.

## 1. Introduction

Truncation methods used in statistical studies, particularly truncated distributions, have received significant attention recently. Truncated distributions have demonstrated their sufficiency and flexibility in data processing. The development of classical distributions has attracted considerable attention from researchers over the past decades. These researchers have contributed to numerous techniques and methods, including generative families, generalization, extension, hybridization, and truncation of distributions. These techniques have contributed to adding highly flexible properties to new distributions, particularly in data modeling. We review some of the most important of these techniques here: the T-X system for generating continuous distributions, introduced by Ref. 1, a new family named Weibull-G was presented by Ref. 2, by relying on the truncation method, a new family of continuous distributions was created by Ref. 3, the Gamma-Weibull-G family distributions developed by Ref. 4, an extended of Weibull-G family of distributions created by Ref. 5, a truncated left and right T-X of generalized families of distributions introduced by Ref. 6, truncated Muth generated (NTM-G) family presented by Ref. 7, and right truncated X-Gamma-G of distributions was presented by Ref. 8. The approach of [0,1] right truncated was applied within different distributions see Refs. 9– 11. In addition, merging distributions has also attracted significant interest from researchers who are interested in generating new distributions. Various methods and techniques have been developed to combine two or more distributions. By using the tail distribution functions, the exponential-Weibull distribution was created by Ref. 12, in keeping with same technique Serial Weibull-Rayleigh distribution has been presented by Ref. 13, a new mixture distribution was come out by mixing three distributions, ^14^ inverse of exponential-Rayleigh proposed by Ref. 15, generalized exponential-Rayleigh introduced by Ref. 16, and Alpha power exponential Weibull model was developed by Ref. 17, with that if *X*~ *ER*( δ,λ ), within *δ* and *λ* are both scale parameters, the (CDF) and (PDF) are given as following.

$$F_{\mathrm{ER}}\left(x;\delta ,\lambda \right)=1-e^{-\left(\mathrm{\delta x}+\frac{\lambda }{2}x^{2}\right)},x,\delta ,\lambda >0$$ (1)

$$f_{\mathrm{ER}}\left(x;\delta ,\lambda \right)=\left(\delta +\mathrm{\lambda x}\right)e^{-\left(\mathrm{\delta x}+\frac{\lambda }{2}x^{2}\right)}$$ (2)

In statistics, a truncated distribution is known as a conditional distribution, where the condition for obtaining it lies in determining the domain of the parent distribution. Determining the domain can be either by restricting the range from above (right truncated), or restricting it from below (left truncated), or restricting it from both sides (interval truncated).

Based on the above, we present here a new distribution coming from truncating the exponential Rayleigh distribution. This distribution is a combination of two distributions that are considered special cases of the Weibull distribution used, is highly effective in modeling data, especially lifetime data. In what is coming, we will shed light on the mathematical construction of statistical functions and properties of the distribution. Furthermore, estimating parameters and applying it to real data and a simulated study.

## 2. [0,1] – Right truncated exponential-Rayleigh distribution

Recently, as a result of the emergence of data of the types of boundaries, percentages, ratios, and rates within the boundary interval, which can be seen in fields such as engineering, economics, and pathology, which deal with infection and mortality rates, the literature on truncated distributions has been rapidly increasing see Refs. 18– 21. First, to drive the (CDF) and (PDF) of [0,1]-RTERD with general forms that are associated with Eqns (1) and (2) are:

$$G_{\text{RTER}}\left(x;\delta ,\lambda \right)=\frac{F_{\mathrm{ER}}\left(x;\delta ,\lambda \right)}{F_{\mathrm{ER}}\left(1;\delta ,\lambda \right)},0\leq x\leq 1,\delta ,\lambda >0$$ (3)

$$g_{\text{RTER}}\left(x;\delta ,\lambda \right)=\frac{f_{\mathrm{ER}}\left(x;\delta ,\lambda \right)}{F_{\mathrm{ER}}\left(1;\delta ,\lambda \right)}$$ (4)

Now, if a random variable *X*~[0,1]-RTERD ( δ,λ ), the corresponding (CDF) and (PDF) according to Eqns (3) and (4) are:

$$G_{\text{RTER}}\left(x;\delta ,\lambda \right)=\frac{1-e^{-\left(\mathrm{\delta x}+\frac{\lambda }{2}x^{2}\right)}}{1-e^{-\left(\delta +\frac{\lambda }{2}\right)}},0\leq x\leq 1,\delta ,\lambda >0$$ (5)

$$g_{\text{RTER}}\left(x;\delta ,\lambda \right)=\frac{\left(\delta +\mathrm{\lambda x}\right)e^{-\left(\mathrm{\delta x}+\frac{\lambda }{2}x^{2}\right)}}{1-e^{-\left(\delta +\frac{\lambda }{2}\right)}}$$ (6)

Considering that $G_{\text{RTER}}\left(x;\delta ,\lambda \right)=1$ for x>1 ,

The survival and hazard functions associated with Eqs (5) and (6), respectively, can be given by the following formulas:

$$S_{\text{RTER}}\left(x;\delta ,\lambda \right)=\frac{e^{-\left(\mathrm{\delta x}+\frac{\lambda }{2}x^{2}\right)}-e^{-\left(\delta +\frac{\lambda }{2}\right)}}{1-e^{-\left(\delta +\frac{\lambda }{2}\right)}}$$ (7)

$$h_{\text{RTER}}\left(x;\delta ,\lambda \right)=\frac{\left(\delta +\mathrm{\lambda x}\right)e^{-\left(\mathrm{\delta x}+\frac{\lambda }{2}x^{2}\right)}}{e^{-\left(\mathrm{\delta x}+\frac{\lambda }{2}x^{2}\right)}-e^{-\left(\delta +\frac{\lambda }{2}\right)}}$$ (8)

## 3. Methodology and mathematical properties

This section focuses on examining the shapes and mathematical properties of the [ 0,1]−RTER D.

### 3.1 The shapes of [0,1]−RTERD

This section discusses the form of the distribution represented by calculating the limits of pdf and hazard functions as they approach to zero and infinity.

$$\lim_{x\to 0}g_{\text{RTER}}\left(x;\delta ,\lambda \right)=\lim_{x\to 0}\left(\frac{\left(\delta +\lambda x\right)e^{-\left(\mathrm{\delta x}+\frac{\lambda }{2}x^{2}\right)}}{1-e^{-\left(\delta +\frac{\lambda }{2}\right)}}\right)=\frac{\delta }{1-e^{-\left(\delta +\frac{\lambda }{2}\right)}}$$

$$\lim_{x\to \infty }g_{\text{RTER}}\left(x;\delta ,\lambda \right)={\left(1-e^{-\left(\delta +\frac{\lambda }{2}\right)}\right)}^{-1}\lim_{x\to \infty }\left(\frac{\delta +\lambda x}{e^{\left(\mathrm{\delta x}+\frac{\lambda }{2}x^{2}\right)}}\right)={\left(1-e^{-\left(\delta +\frac{\lambda }{2}\right)}\right)}^{-1}\lim_{x\to \infty }\left(\frac{\lambda }{\left(\delta +\lambda x\right)e^{\left(\mathrm{\delta x}+\frac{\lambda }{2}x^{2}\right)}}\right)={\left(1-e^{-\left(\delta +\frac{\lambda }{2}\right)}\right)}^{-1}\left(\frac{\lambda }{\infty }\right)=0$$

$$\lim_{x\to 0}h_{\text{RTER}}\left(x;\delta ,\lambda \right)=\lim_{x\to 0}\left(\frac{\left(\delta +\lambda x\right)e^{-\left(\mathrm{\delta x}+\frac{\lambda }{2}x^{2}\right)}}{e^{-\left(\mathrm{\delta x}+\frac{\lambda }{2}x^{2}\right)}-e^{-\left(\delta +\frac{\lambda }{2}\right)}}\right)=\frac{\delta }{1-e^{-\left(\delta +\frac{\lambda }{2}\right)}}$$

$$\lim_{x\to \infty }h_{\text{RTER}}\left(x;\delta ,\lambda \right)=\lim_{x\to \infty }\left(\frac{\left(\delta +\lambda x\right)e^{-\left(\mathrm{\delta x}+\frac{\lambda }{2}x^{2}\right)}}{e^{-\left(\mathrm{\delta x}+\frac{\lambda }{2}x^{2}\right)}-e^{-\left(\delta +\frac{\lambda }{2}\right)}}\right)=\lim_{x\to \infty }\left(\frac{\delta +\lambda x}{1-e^{\delta \left(x-1\right)+\frac{\lambda }{2}\left(x^{2}-1\right)}}\right)=\lim_{x\to \infty }\left(\frac{\delta }{-\left(\delta +\lambda x\right)e^{\delta \left(x-1\right)+\frac{\lambda }{2}\left(x^{2}-1\right)}}\right)=\frac{-\delta }{\infty }=0\;$$

**Figure 1.**
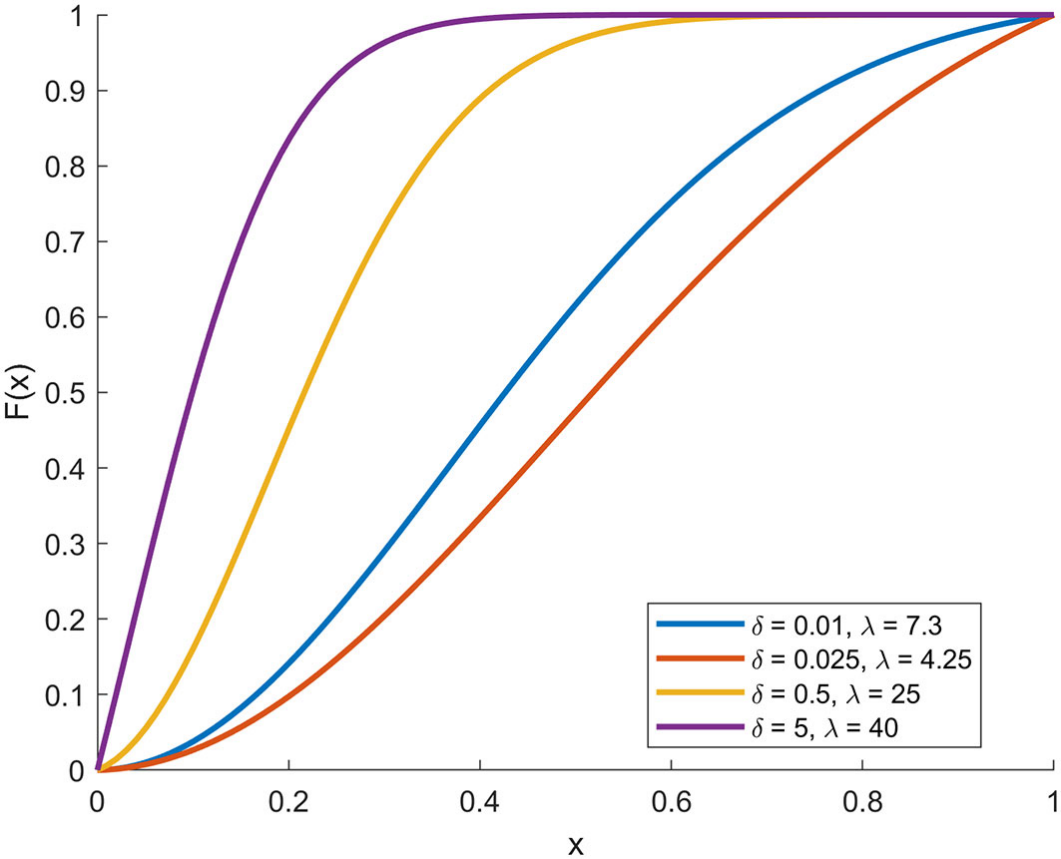
Description of the figure of cdf of [0,1]-RTERD.

**Figure 2.**
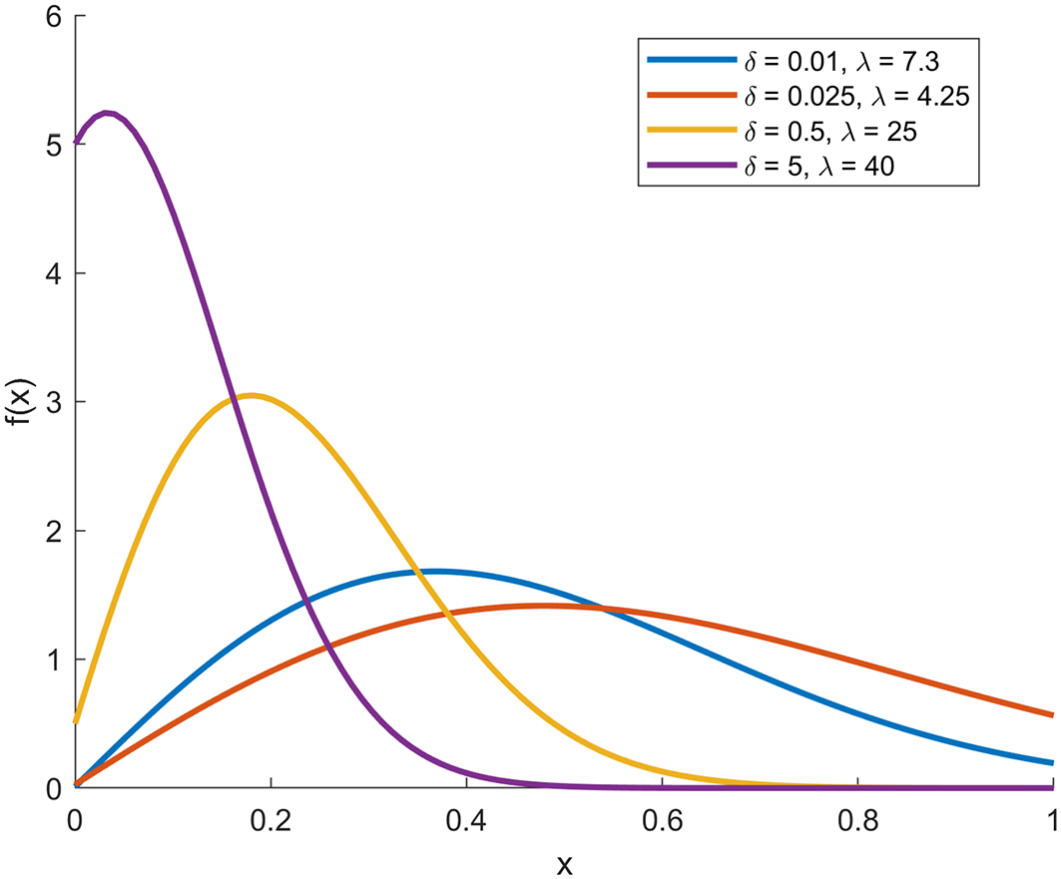
Description of the figure pdf of [0,1]-RTERD.

**Figure 3.**
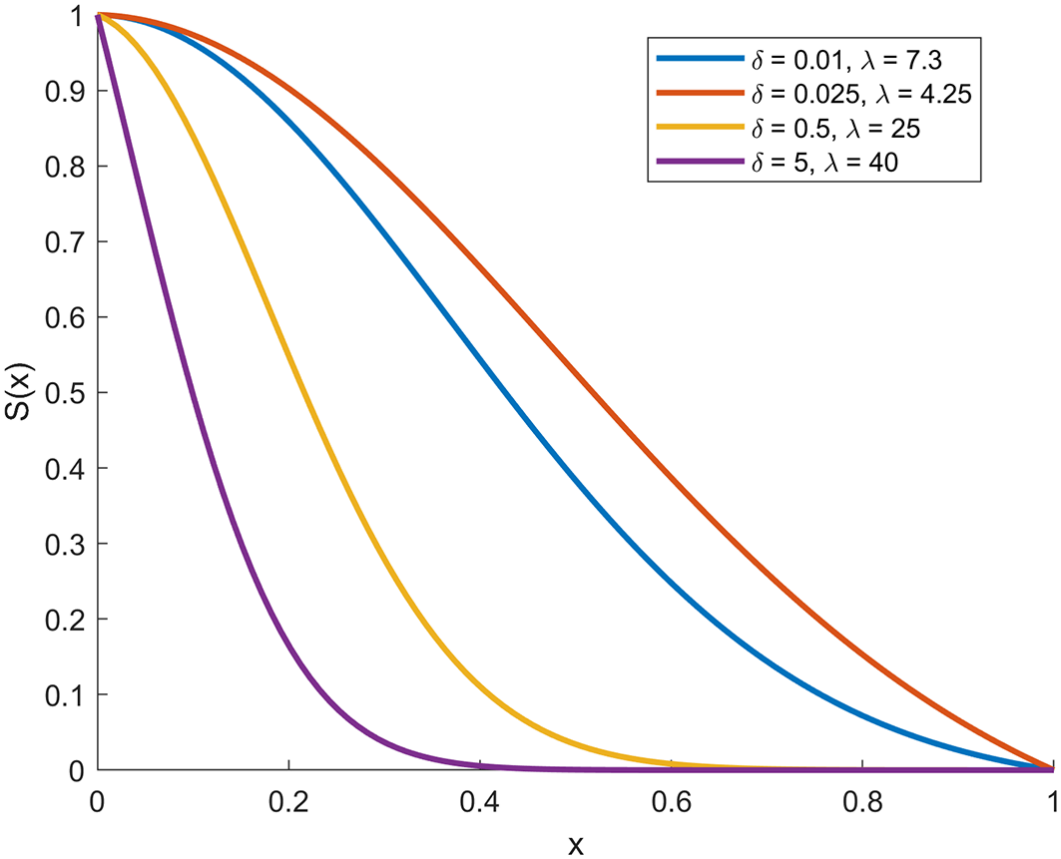
Description of the figure *S* ( *x*) of [0,1]-RTERD.

**Figure 4.**
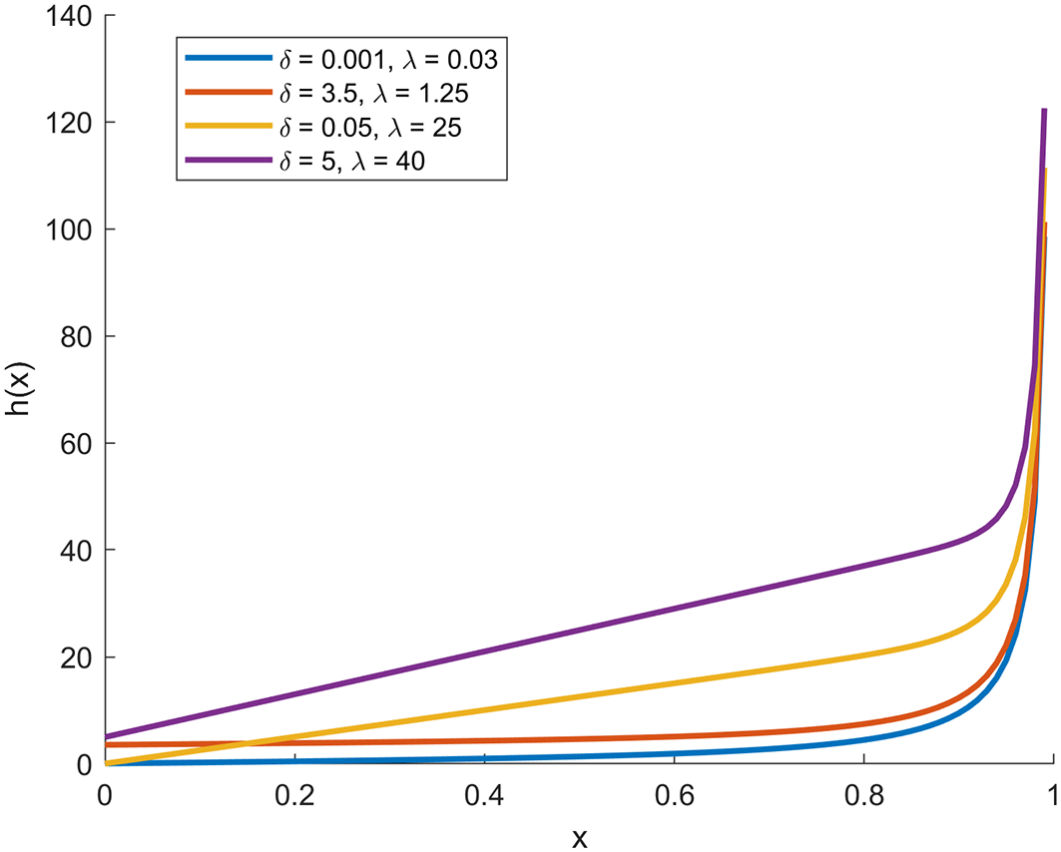
Description of the figure *h* ( *x*) of [0,1]-RTERD.

In the following, through Figures 1, 2, 3, and 4, we present plots (CDF), (PDF), *S* ( *x*), and *h* ( *x*) functions with deferent parameters values.

In the figure above, the four plots represent the basic statistical properties of proposed distribution under different parameters values.

Figure 1 shows the CDF plot, where all curves start from zero and gradually increase towards one as ( *x*) increases. The results show that increasing the value of ( δ ) accelerates the accumulation of probability, while increasing (λ) slows it down, reflecting the influence of these two parameters in determining the shape and spread of [0,1]-RTERD.

Figure 2 shows the PDF plot with demonstrates that changing the parameters leads to a clear difference in the shape of the distribution and the position of its peak. The peaks tend towards smaller values of ( *x*) as ( δ ) increases, indicating a leftward bias in the distribution.

Figure 3 shows the survival function ( *S*( *x*)), which gradually decreases as *x* increases. It is evident that increasing δ decreases the probability of survival, while increasing λ slows the rate of decrease.

Figure 4 shows the hazard function ( *H*( *x*)), which exhibits an increasing pattern indicating a rising probability of failure or risk as x increases. It is also evident that higher λ values lead to a higher risk rate at higher *x* values.

Overall, these results demonstrate the flexibility of [0,1]-RTERD in representing different behaviors of probability functions and its ability to characterize data of varying shapes by considering the influence of the parameters on the shape of [0,1]-RTERD, its centering, and the behavior of the risk function.

### 3.2 Moments, skewness and kurtosis

Moments are considered one of the most important statistical properties, as they are the basis for some other properties, such as E(x),var(x), skewness (S.C), and kurtosis (K.C). The moments of [0,1]-RTERD are;

$$M_{r}^{\prime }=E\left(x^{r}\right)=\int_{0}^{1}x^{r}∙g_{\text{RTER}}\left(x;\delta ,\lambda \right)\;\mathrm{dx}=\frac{1}{1-e^{-\left(\delta +\frac{\lambda }{2}\right)}}\int_{0}^{1}x^{r}∙\left(\delta +\lambda x\right)e^{-\left(\mathrm{\delta x}+\frac{\lambda }{2}x^{2}\right)}\;\mathrm{dx}=\frac{1}{1-e^{-\left(\delta +\frac{\lambda }{2}\right)}}\int_{0}^{1}x^{r}∙\left(\delta +\lambda x\right)e^{-\mathrm{\delta x}}e^{\frac{-\lambda }{2}x^{2}}\;\mathrm{dx}$$

$$e^{\frac{-\lambda }{2}x^{2}}=\sum_{i=0}^{\infty }\frac{{\left(-1\right)}^{i}\lambda^{i}}{2^{i}i!}x^{2i}$$

$$M_{r}^{\prime }=\frac{\sum_{i=0}^{\infty }\frac{{\left(-1\right)}^{i}\lambda^{i}}{2^{i}i!}}{1-e^{-\left(\delta +\frac{\lambda }{2}\right)}}\int_{0}^{1}x^{r+2i}∙\left(\delta +\lambda x\right)e^{-\mathrm{\delta x}}\;\mathrm{dx}$$

Let $y=\mathrm{\delta x}⇨x=\frac{y}{\delta }⇨\mathrm{dx}=\frac{\mathrm{dy}}{\delta }$

$$\begin{array}{l} M_{r}^{\prime }=\frac{\sum_{i=0}^{\infty }\frac{{\left(-1\right)}^{i}\lambda^{i}}{2^{i}i!}}{1-e^{-\left(\delta +\frac{\lambda }{2}\right)}}\int_{0}^{1}x^{r+2i}∙\left(\delta +\lambda x\right)e^{-y}\;\mathrm{dx}\\ \;=\frac{\sum_{i=0}^{\infty }\frac{{\left(-1\right)}^{i}\lambda^{i}}{2^{i}i!}}{1-e^{-\left(\delta +\frac{\lambda }{2}\right)}}\int_{0}^{1}\frac{y^{r+2i}}{\delta^{r+2i+1}}∙\left(\delta +\frac{\lambda }{\delta }y\right)e^{-y}\;\mathrm{dy}\\ \;=\frac{\sum_{i=0}^{\infty }\frac{{\left(-1\right)}^{i}\lambda^{i}}{2^{i}i!}}{\delta^{r+2i+1}\left(1-e^{-\left(\delta +\frac{\lambda }{2}\right)}\right)}\left(\delta \int_{0}^{1}y^{r+2i}e^{-y}\;\mathrm{dy}+\frac{\lambda }{\delta }\int_{0}^{1}y^{r+2i+1}e^{-y}\;\mathrm{dy}\right) \end{array}$$

$$M_{r}^{\prime }=\frac{1}{\delta^{r+2i+1}\left(1-e^{-\left(\delta +\frac{\lambda }{2}\right)}\right)}\sum_{i=0}^{\infty }\frac{{\left(-1\right)}^{i}\lambda^{i}}{2^{i}i!}\left(\delta \;\gamma \left(r+2i+1,1\right)+\frac{\lambda }{\delta }\;\gamma \left(r+2i+2,1\right)\right)$$ (9)

Remark. If a random variable *X*~[0,1]-RTER( δ,λ ), the following are satisfy.

$$M_{1}^{\prime }=E\left(x\right)=\frac{1}{\delta^{r+2i+1}\left(1-e^{-\left(\delta +\frac{\lambda }{2}\right)}\right)}\sum_{i=0}^{\infty }\frac{{\left(-1\right)}^{i}\lambda^{i}}{2^{i}i!}\left(\delta \gamma \left(2i+2,1\right)+\frac{\lambda }{\delta }\gamma \left(2i+3,1\right)\right)$$ (10)

$$M_{2}^{\prime }=\frac{1}{\delta^{r+2i+1}\left(1-e^{-\left(\delta +\frac{\lambda }{2}\right)}\right)}\sum_{i=0}^{\infty }\frac{{\left(-1\right)}^{i}\lambda^{i}}{2^{i}i!}\left(\delta \gamma \left(2i+3,1\right)+\frac{\lambda }{\delta }\gamma \left(2i+4,1\right)\right)$$ (11)

$$M_{3}^{\prime }=\frac{1}{\delta^{r+2i+1}\left(1-e^{-\left(\delta +\frac{\lambda }{2}\right)}\right)}\sum_{i=0}^{\infty }\frac{{\left(-1\right)}^{i}\lambda^{i}}{2^{i}i!}\left(\delta \gamma \left(2i+4,1\right)+\frac{\lambda }{\delta }\gamma \left(2i+5,1\right)\right)$$ (12)

$$M_{4}^{\prime }=\frac{1}{\delta^{r+2i+1}\left(1-e^{-\left(\delta +\frac{\lambda }{2}\right)}\right)}\sum_{i=0}^{\infty }\frac{{\left(-1\right)}^{i}\lambda^{i}}{2^{i}i!}\left(\delta \gamma \left(2i+5,1\right)+\frac{\lambda }{\delta }\gamma \left(2i+6,1\right)\right)$$ (13)

$$\text{var}\left(x\right)=M_{2}^{\prime }-{\left(M_{1}^{\prime }\right)}^{2}$$ (14)

S.C=M3′(M2′)32 (15)

$$\mathrm{C.K}=\frac{M_{4}^{\prime }}{{\left(M_{2}^{\prime }\right)}^{2}}-3$$ (16)

Table 1 shows the moment values when *r* = 1, 2, 3, and 4, as well as the values of variance, skewness, and kurtosis, where the data values were confined to the interval [0,1], which represents the integral limits for the moments.

**Table 1.** 1 ^st^-4 ^th^ moments, skewness, kurtosis, and variance values for x∈[0,1].

*δ*	*λ*	$M_{1}^{\prime }$	$M_{2}^{\prime }$	$M_{3}^{\prime }$	$M_{4}^{\prime }$	K.C	S.C	var
1.5	2.5	0.3718	0.2036	0.1331	0.0964	-0.6758	1.4489	0.0654
2.5	1.5	0.3028	0.1491	0.0917	0.0639	-0.1264	1.5924	0.0574
2.75	0.25	0.2945	0.1453	0.0903	0.0637	0.0166	1.6304	0.0586
3.75	1.25	0.2355	0.0980	0.0538	0.0347	0.6079	1.7535	0.0426
0.75	3.25	0.4415	0.2617	0.1788	0.1330	1.3355	-1.0585	0.0669

### 3.3 Moment-generating function

It is one of the important properties that we obtain and is given by the following formula:

$$\begin{array}{c} M_{X}\left(t\right)=E\left(e^{\mathrm{xt}}\right)=\int_{0}^{1}e^{\mathrm{xt}}∙g_{\text{RTER}}\left(x;\delta ,\lambda \right)\;\mathrm{dx}\\ \;=\frac{1}{1-e^{-\left(\delta +\frac{\lambda }{2}\right)}}\int_{0}^{1}e^{\mathrm{xt}}∙\left(\delta +\lambda x\right)e^{-\left(\mathrm{\delta x}+\frac{\lambda }{2}x^{2}\right)}\;\mathrm{dx}\\ \;=\frac{1}{1-e^{-\left(\delta +\frac{\lambda }{2}\right)}}\int_{0}^{1}\left(\delta +\lambda x\right)e^{-\left(\delta +t\right)x-\frac{\lambda }{2}x^{2}}\;\mathrm{dx}\\ \;=\frac{\sum_{i=0}^{\infty }\frac{{\left(-1\right)}^{i}\lambda^{i}}{2^{i}i!}}{1-e^{-\left(\delta +\frac{\lambda }{2}\right)}}\int_{0}^{1}x^{2i}\left(\delta +\lambda x\right)e^{-\left(\delta +t\right)x}\;\mathrm{dx} \end{array}$$

Let $y=\left(\delta +t\right)x⇨x=\frac{y}{\delta +t}⇨\mathrm{dx}=\frac{\mathrm{dy}}{\delta +t}$

$$M_{X}\left(t\right)=\frac{\sum_{i=0}^{\infty }\frac{{\left(-1\right)}^{i}\lambda^{i}}{2^{i}i!}}{{\left(\delta +t\right)}^{2i+1}\left(1-e^{-\left(\delta +\frac{\lambda }{2}\right)}\right)}\int_{0}^{1}y^{2i}\left(\delta +\frac{\lambda }{y}\delta +t\right)e^{-y}\;\mathrm{dy}=\frac{\sum_{i=0}^{\infty }\frac{{\left(-1\right)}^{i}\lambda^{i}}{2^{i}i!}}{{\left(\delta +t\right)}^{2i+1}\left(1-e^{-\left(\delta +\frac{\lambda }{2}\right)}\right)}\left(\delta \int_{0}^{1}y^{2i}e^{-y}\;\mathrm{dy}+\frac{\lambda }{\delta +t}\int_{0}^{1}y^{2i+1}e^{-y}\;\mathrm{dy}\right)$$

$$M_{X}\left(t\right)=\frac{\sum_{i=0}^{\infty }\frac{{\left(-1\right)}^{i}\lambda^{i}}{2^{i}i!}}{{\left(\delta +t\right)}^{2i+1}\left(1-e^{-\left(\delta +\frac{\lambda }{2}\right)}\right)}\left(\delta \gamma \left(2i+1,1\right)+\frac{\lambda }{\delta +t}\gamma \left(2i+2,1\right)\right)$$ (17)

### 3.4 Quantile function

$$Q\left(u\right)=G^{-1}\left(u\right),u\in \left(0,1\right)$$

$$\;\frac{1-e^{-\left(\mathrm{\delta x}+\frac{\lambda }{2}x^{2}\right)}}{1-e^{-\left(\delta +\frac{\lambda }{2}\right)}}=u$$

$$e^{-\left(\mathrm{\delta x}+\frac{\lambda }{2}x^{2}\right)}=1-u\left(1-e^{-\left(\delta +\frac{\lambda }{2}\right)}\right)$$

$$\mathrm{\delta x}+\frac{\lambda }{2}x^{2}=-\ln\left(1-u\left(1-e^{-\left(\delta +\frac{\lambda }{2}\right)}\right)\right)$$

$$\frac{\lambda }{2}x^{2}+\mathrm{\delta x}+\ln\left(1-u\left(1-e^{-\left(\delta +\frac{\lambda }{2}\right)}\right)\right)=0$$

$$Q\left(u\right)=x_{u}=\frac{1}{\lambda }\left(-\delta \pm \sqrt{\delta^{2}-2\lambda \text{ln}\left(1-u\left(1-e^{-\left(\delta +\frac{\lambda }{2}\right)}\right)\right)}\right)$$ (18)

A spatial case of eqn. (18), the median of [0,1]-RTERD is verified when *u* = 0.5.

$$Q\left(0.5\right)=x_{0.5}=\frac{1}{\lambda }\left(-\delta \pm \sqrt{\delta^{2}-2\lambda \text{ln}\left(1-0.5\left(1-e^{-\left(\delta +\frac{\lambda }{2}\right)}\right)\right)}\right)$$ (19)

### 3.5 Rėnyi entropy

One of the most important properties that play a fundamental role in extracting information is the randomness measure defined as follows ^22^:

$${\text{₮}}_{\text{RTER}}\left(\eta \right)=\frac{1}{1-\eta }\text{log}\left(\int_{0}^{1}g_{\text{RTER}}^{\eta }\left(x\right)\mathrm{dx}\right)=\frac{1}{1-\eta }\text{log}\left(\frac{1}{{\left(1-e^{-\left(\delta +\frac{\lambda }{2}\right)}\right)}^{\eta }}\int_{0}^{1}{\left(\delta +\mathrm{\lambda x}\right)}^{\eta }e^{-\eta \left(\mathrm{\delta x}+\frac{\lambda }{2}x^{2}\right)}\mathrm{dx}\right)$$

$${\left(\delta +\lambda x\right)}^{\eta }=\sum_{j=0}^{\infty }\left(\begin{array}{c} \eta \\ j \end{array}\right)\delta^{\eta -j}\lambda^{j}x^{j}$$

$$e^{\frac{-\eta \lambda }{2}x^{2}}=\sum_{p=0}^{\infty }\frac{{\left(-1\right)}^{p}\eta^{p}\lambda^{p}}{2^{p}p!}x^{2p}$$

$${\text{₮}}_{\text{RTER}}\left(\eta \right)=\frac{1}{1-\eta }\text{log}\left(\frac{\sum_{j}^{\infty }\sum_{p}^{\infty }\left(\begin{array}{c} \eta \\ j \end{array}\right)\frac{{\left(-1\right)}^{p}\eta^{p}\lambda^{p}}{2^{p}p!}}{{\left(1-e^{-\left(\delta +\frac{\lambda }{2}\right)}\right)}^{\eta }}\int_{0}^{1}x^{2p+j}e^{-\mathrm{\eta \delta x}}\mathrm{dx}\right)$$

Let $y=\mathrm{\eta \delta x}⇨x=\frac{y}{\eta \delta }⇨\mathrm{dx}=\frac{\mathrm{dy}}{\eta \delta }$

$${\text{₮}}_{\text{RTER}}\left(\eta \right)=\frac{1}{1-\eta }\text{log}\left(\frac{\sum_{j}^{\infty }\sum_{p}^{\infty }\left(\begin{array}{c} \eta \\ j \end{array}\right)\frac{{\left(-1\right)}^{p}\eta^{p}\lambda^{p}}{2^{p}p!{\left(\eta \delta \right)}^{2p+j+1}}}{{\left(1-e^{-\left(\delta +\frac{\lambda }{2}\right)}\right)}^{\eta }}\int_{0}^{1}y^{2p+j}e^{-y}\mathrm{dy}\right)$$

$${\text{₮}}_{\text{RTER}}\left(\eta \right)=\frac{1}{1-\eta }\text{log}\left(\frac{\sum_{j}^{\infty }\sum_{p}^{\infty }\left(\begin{array}{c} \eta \\ j \end{array}\right)\frac{{\left(-1\right)}^{p}\eta^{p}\lambda^{p}}{2^{p}p!{\left(\eta \delta \right)}^{2p+j+1}}}{{\left(1-e^{-\left(\delta +\frac{\lambda }{2}\right)}\right)}^{\eta }}\gamma \left(2p+j+1,1\right)\right)$$ (20)

### 3.6 Order statistic

Let $x_{1:K}\leq x_{2:K}\leq x_{3:K}\leq \ldots \leq x_{K:K}$ be the order statistics of a random sample $x_{1},x_{2},\ldots ,x_{K}$ of size *k* from [0,1]-RTERD. The PDF for order statistics is as follows:

$$\begin{array}{l} g_{P,K}\left(x\right)=\frac{K!}{\left(P-1\right)!\left(K-P\right)!}{\left(G_{\text{RTER}}\left(x;\delta ,\lambda \right)\right)}^{P-1}{\left(1-G_{\text{RTER}}\left(x;\delta ,\lambda \right)\right)}^{K-P}g_{\text{RTER}}\left(x;\delta ,\lambda \right)\\ \;=\text{₭}{\left(\frac{1-e^{-\left(\mathrm{\delta x}+\frac{\lambda }{2}x^{2}\right)}}{1-e^{-\left(\delta +\frac{\lambda }{2}\right)}}\right)}^{P-1}{\left(\frac{e^{-\left(\mathrm{\delta x}+\frac{\lambda }{2}x^{2}\right)}-e^{-\left(\delta +\frac{\lambda }{2}\right)}}{1-e^{-\left(\delta +\frac{\lambda }{2}\right)}}\right)}^{K-P}\frac{\left(\delta +\lambda x\right)e^{-\left(\mathrm{\delta x}+\frac{\lambda }{2}x^{2}\right)}}{1-e^{-\left(\delta +\frac{\lambda }{2}\right)}} \end{array}$$

Where $\text{₭}=\frac{K!}{\left(P-1\right)!\left(K-P\right)!}$

$$g_{P,K}\left(x\right)=\text{₭}\frac{{\left(1-e^{-\left(\mathrm{\delta x}+\frac{\lambda }{2}x^{2}\right)}\right)}^{P-1}{\left(e^{-\left(\mathrm{\delta x}+\frac{\lambda }{2}x^{2}\right)}-e^{-\left(\delta +\frac{\lambda }{2}\right)}\right)}^{K-P}\left(\left(\delta +\mathrm{\lambda x}\right)e^{-\left(\mathrm{\delta x}+\frac{\lambda }{2}x^{2}\right)}\right)}{{\left(1-e^{-\left(\delta +\frac{\lambda }{2}\right)}\right)}^{K}}$$ (21)

The minimum order statistics of PDF, when P=1 , and the maximum value among the order statistics, when P=K , of the [0,1]-RTERD, is given in the following form:

$$g_{1,K}\left(x\right)=\text{₭}\frac{{\left(e^{-\left(\mathrm{\delta x}+\frac{\lambda }{2}x^{2}\right)}-e^{-\left(\delta +\frac{\lambda }{2}\right)}\right)}^{K-1}\left(\left(\delta +\mathrm{\lambda x}\right)e^{-\left(\mathrm{\delta x}+\frac{\lambda }{2}x^{2}\right)}\right)}{{\left(1-e^{-\left(\delta +\frac{\lambda }{2}\right)}\right)}^{K}}$$ (22)

$$g_{K,K}\left(x\right)=\text{₭}\frac{{\left(1-e^{-\left(\mathrm{\delta x}+\frac{\lambda }{2}x^{2}\right)}\right)}^{K-1}\left(\left(\delta +\mathrm{\lambda x}\right)e^{-\left(\mathrm{\delta x}+\frac{\lambda }{2}x^{2}\right)}\right)}{{\left(1-e^{-\left(\delta +\frac{\lambda }{2}\right)}\right)}^{K}}$$ (23)

## 4. Maximum likelihood estimation

In order to estimate the parameters, MLE applied to estimate the two parameters of [0,1]-RTERD. Let $x_{1},x_{2},\cdots x_{n}$ be random samples that are distributed as [0,1]-RTERD, then the likelihood function is:

$$\begin{array}{l} L\left(x_{1},x_{2},\cdots ,x_{n};\delta ,\lambda \right)=\prod_{i=1}^{n}g_{\text{RTER}}\left(x_{i};\delta ,\lambda \right)=\prod_{i=1}^{n}\frac{\left(\delta +\lambda x_{i}\right)e^{-\left(\delta x_{i}+\frac{\lambda }{2}{x_{i}}^{2}\right)}}{1-e^{-\left(\delta +\frac{\lambda }{2}\right)}}\\ \;={\left(1-e^{-\left(\delta +\frac{\lambda }{2}\right)}\right)}^{-n}e^{-\sum_{i=1}^{n}\left(\delta x_{i}+\frac{\lambda }{2}{x_{i}}^{2}\right)}\prod_{i=1}^{n}\left(\delta +\lambda x_{i}\right)\\ \;\hat{L}=\ln\left(L\right)=-\mathrm{nln}\left(1-e^{-\left(\delta +\frac{\lambda }{2}\right)}\right)-\sum_{i=1}^{n}\left(\delta x_{i}+\frac{\lambda }{2}{x_{i}}^{2}\right)+\sum_{i=1}^{n}\ln\left(\delta +\lambda x_{i}\right) \end{array}$$

$$\frac{\partial \hat{L}}{\partial \delta }=\frac{-ne^{-\left(\delta +\frac{\lambda }{2}\right)}}{1-e^{-\left(\delta +\frac{\lambda }{2}\right)}}-\sum_{i=1}^{n}x_{i}+\sum_{i=1}^{n}\frac{1}{\delta +\lambda x_{i}}$$ (24)

$$\frac{\partial \hat{L}}{\partial \lambda }=\frac{-ne^{-\left(\delta +\frac{\lambda }{2}\right)}}{2\left(1-e^{-\left(\delta +\frac{\lambda }{2}\right)}\right)}-\frac{1}{2}\sum_{i=1}^{n}{x_{i}}^{2}+\sum_{i=1}^{n}\frac{x_{i}}{\delta +\lambda x_{i}}$$ (25)

After setting Equations (24) and (25) equal to zero, we obtain nonlinear equations whose solutions are found, representing the estimated parameter values, using MATLAB programming. ^23^

## 5. Simulation

Monte-Carlo simulation applied to examine statistical performance of MLE for [0,1]-RTERD. The evaluation centered on two primary accuracy indicators: bias and mean squared error (MSE). To achieve this, random samples were generated based on Equation (18), using different sizes ( *n* = 20, 30, 50, 100, and 200), and repeated across L = 1000 replications. Parameter estimates were obtained via (MLE), with starting values specified as *δ* = 0.25, λ = 1.5 and δ = 0.1, λ = 0.5. The simulations were implemented using MATLAB (version 2018a).

Table 2 shows the parameter estimates using (MLE) across different sample sizes (20, 30, 50, 100, and 200). The results proved that the accuracy of the estimates gradually improves with increasing sample size, as estimated values approach the true values of the parameters, and their variance decreases, as evidenced by the decline in the MSE, bias, and the mean values for each parameter. In smaller samples, the estimates were less accurate, while they became more reliable and consistent in larger samples. This confirms that the MLE method gains greater accuracy and stability with increasing sample size.

**Table 2.** Average estimate, MSE, and Bias values corresponding to MLE.

Parameter	n	Mean $\left(\hat{\delta }\right)$	Mean $\left(\hat{\lambda }\right)$	MSE $\left(\hat{\delta }\right)$	Bias $\left(\hat{\delta }\right)$	MSE $\left(\hat{\lambda }\right)$	Bias $\left(\hat{\lambda }\right)$
δ=0.25 λ=1.5	20	0.2568	1.3027	0.0314	0.0068	0.4748	-0.1973
	30	0.2586	1.3092	0.0261	0.0086	0.4639	-0.1908
	50	0.2496	1.3618	0.0191	-0.0004	0.4085	-0.1382
	100	0.2386	1.4106	0.0121	-0.0114	0.3456	-0.0894
	200	0.2371	1.4207	0.0062	-0.0129	0.2976	-0.0793
δ=0.1 λ=0.5	20	0.1438	0.6666	0.0198	0.0438	0.1377	0.1666
	30	0.1271	0.6387	0.0126	0.0271	0.1275	0.1387
	50	0.1193	0.6126	0.0085	0.0193	0.1228	0.1126
	100	0.1058	0.6052	0.0041	0.0058	0.1163	0.1052
	200	0.1018	0.5988	0.0024	0.0018	0.1043	0.0988

**Figure 5.**
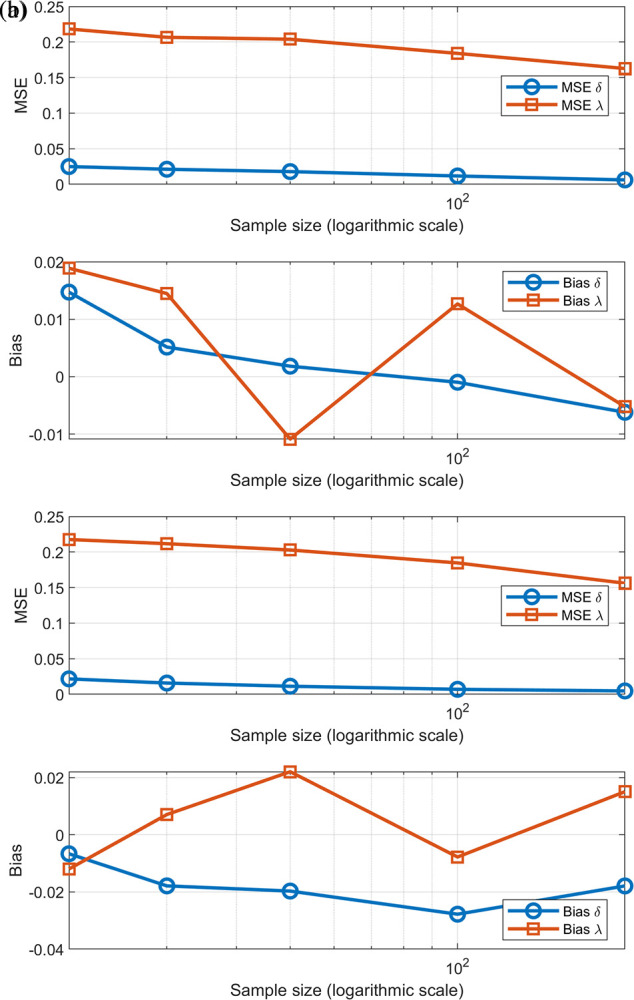
(a) Description of MSE and Bias Estimation Performances [0,1]-RTERD of (δ = 0.25, λ = 1.5). (b) Description of MSE and Bias Estimation Performances [0,1]-RTERD of (δ = 0.1, λ = 0.5).

**Figure 6.**
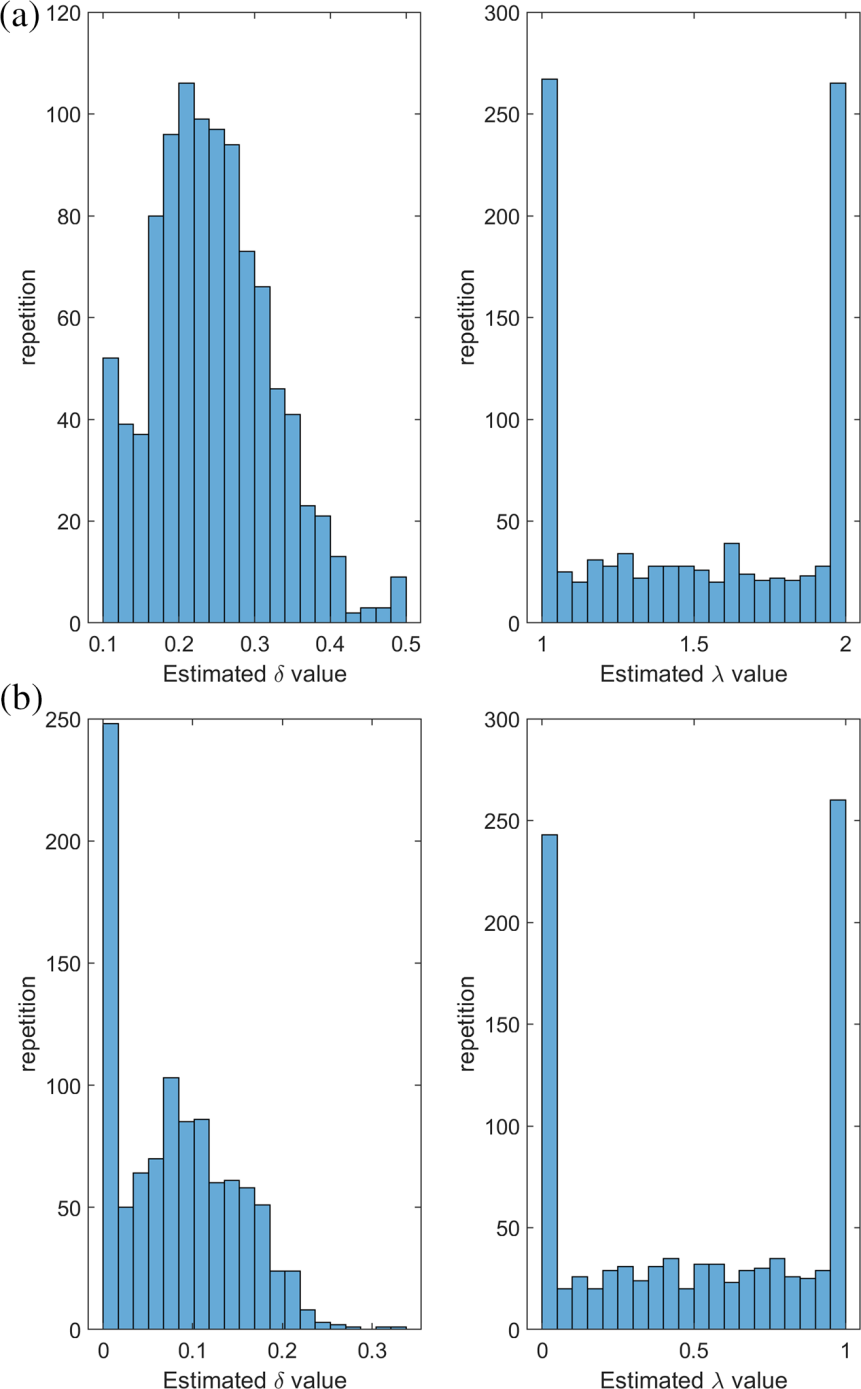
(a) Description of the Histogram of estimate values of δ = 0.25, λ = 1.5 of [0,1]-RTERD. (b) Description of the Histogram of estimate values of δ = 0.1, λ = 0.5 of [0,1]-RTERD.

Figures 5 & 6 reinforce the content discussed above in terms of what can be observed regarding the values of estimators, error, and bias.

## 6. Application

Information criteria are tools used to demonstrate a distribution’s flexibility and effectiveness in representing realistic data by comparing it to the performance of other distributions. Some of these criteria, such as the $\mathrm{AIC},{\mathrm{AIC}}_{c},\mathrm{BIC},\text{and HQIC}$ , will be applied to a data set representing The data represents the failure time of 50 components ( $10^{3}$ hours), as it is complete data with a rightward skewed nature. These data were utilized by Arshad MZ et al., ^24^ and are presented as follows:

(0.213, 0.275, 0.099, 0.388, 0.497, 0.087, 0.073, 0.089, 0.061, 0.086, 0.089, 0.790, 0.118, 0.075, 0.297, 0.119, 0.299, 0.315, 0.403, 0.192, 0.308, 0.191, 0.909, 0.168, 0.135, 0.314, 0.117, 0.120, 0.183, 0.714, 0.143, 0.374, 0.859, 0.084, 0.089, 0.215, 0.297, 0.992, 0.330, 0.817, 0.298, 0.258, 0.123, 0.815, 0.088, 0.102, 0.203, 0.199, 0.185, 0.257).

**Figure 7.**
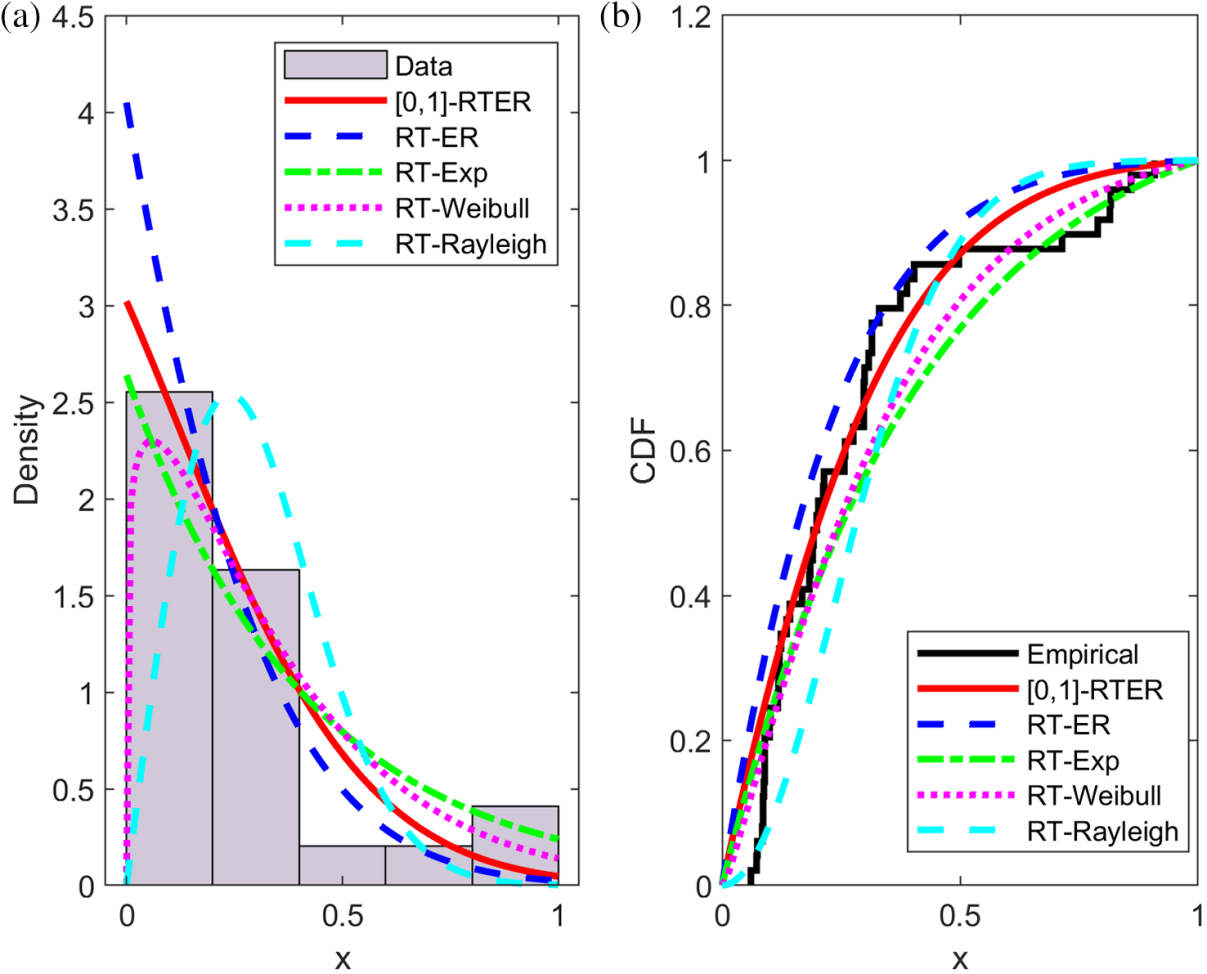
Description of turbochargers data in term of empirical histogram and empirical cumulative [0,1]-RTERD.

Table 3 shows the values of the information criteria and the performance of [0,1]-RTERD compared to the performance of the other models RTED, RTWD, RTRD, and ERD in representing the data above. It is noticeable that the distribution is capable and effective, as shown in the included results. In addition, the [0,1]-RTERD was compared in terms of representing the pdf’s and cdf’s functions for turbochargers data through Figure 7.

**Table 3.** Negative log (L) and information criteria.

Dist.	-log(L)	AIC	BIC	AICc	HIQC
RTER	12.558	29.103	32.928	29.359	30.559
RTE	17.831	39.661	43.485	39.916	41.117
RTW	17.161	39.322	44.102	39.698	41.142
RTR	21.808	43.716	43.812	43.718	43.752
ER	14.818	33.635	37.459	33.890	35.091

Figure 7 show a comparison between the pdf.s and cdf.s for several interpolated [0,1] distributions: RTER, ER, RTE, RTW, and RTR distributions, along with the experimental data.

Figure 7(a) it is evident that [[0,1]-RTER (solid red line) best matches the overall pattern of the sample data, particularly in the initial region of small *x*-values, where its curve closely resembles the experimental density pattern. The other distributions show relative deviations; RTR tends to overestimate small values, while ER and RTE provide less accurate estimates in the tail regions.

Figure 7(b) highlights degree of agreement between the estimated cumulative functions and the experimental function. We observe that the RTER curve best represents data behavior across the entire interval [0,1], outperforming the other distributions, which either exceed the empirical function or deviate from it at the middle and upper values of ( *x*).

Overall, the results confirm that the proposed RTER distribution exhibits greater flexibility and better representation of truncated data compared to the other distributions, reflecting its efficiency in modeling real data within the finite interval [0,1].

## Conclusion

The proposed [0,1]-RTERD offers a flexible generalization of the classical exponential and Rayleigh distributions. This enhancement allows the model to accommodate various shapes of hazard functions, improving its suitability for modeling lifetime and reliability data. The properties of the distribution were systematically derived, and parameter estimation was performed using MLE method. To assess the model’s goodness-of-fit, several information-based criteria were employed, including AIC, ${\mathrm{AIC}}_{c}$ , BIC, and HQIC. The results indicate that the RTERD outperforms traditional models, demonstrating a superior fit to real-world data and highlighting its potential as a robust tool in applied reliability and survival analysis.

## Ethics and consent

Since the study did not include sensitive or personal data or human subjects, there is no ethical or approval requirement for this study.

## Data Availability

Zenodo: Real experimental data supporting this study. https://doi.org/10.5281/zenodo.18071193 or https://github.com/alitm-afk/Application-of-Failure-time-of-50- components. ^24, 25^ The project contains the following underlying data: [Application-of-Failure-time-of-50-components-main.zip] (The Failure Time of 50 Components (10 ^3^ hours) Information criteria are tools used to demonstrate a distribution’s flexibility and effectiveness in representing realistic data by comparing it to the performance of other distributions. Some of these criteria, such as the AIC, AIC_c, BIC, and HQIC, will be applied to a data set representing, the data represents the failure time of 50 components (10 ^3^ hours), as it is complete data with a rightward skewed nature). (contains the raw experimental data used to generate Figure 7 and the results reported in Table 3). Zenodo: Simulation and numerical data used in this study. https://doi.org/10.5281/zenodo.18119198 ^26^ This project contains the following extended data:
1. Figures(1,2,3,4)_dataset.xlsx (data used to generate Figures 1–4). 2. simulation_data.xlsx (simulation results used to generate figures 5 and 6 and results used in Table 2). 3. moments, skewness, kurtosis, and variance.docx (descriptive statistics and calculations associated with the simulation data and results used in Table 1). Data are available under the terms of the Creative Commons Attribution 4.0 International license (CC-BY 4.0).
