# Supplementary material for: Modified New [0, 1] - Right Truncated Exponential Rayleigh Distribution Structure and Application
Source: F1000Res. 2026 Jan 21;15:102. [Version 1] doi: 10.12688/f1000research.174089.1 (PMC13202200; doi:10.12688/f1000research.174089.1)
Supplement: Peer review history

# Reviewer report: Reviewer response for version 1

**DOI:** 10.5256/f1000research.191964.r470691

**Published:** 2026-05-25

**Authors:** Qasim Nasir Husain

**Affiliations:** 1 Tikrit University, Baghdad, Iraq

My review of the research paper reveals the following: a serious proposal to expand and merge two basic distributions to obtain a new family, allowing the researcher to study the general and fundamental characteristics of the proposed family. There is also effective manipulation and modification of the parameters of the merged distributions to achieve greater flexibility. The results are supported by a simulation model, which is a strength of the work. The researcher needs to elaborate further in the conclusion to better illustrate the results and the subsequent analysis.

Is the work clearly and accurately presented and does it cite the current literature?

Yes

If applicable, is the statistical analysis and its interpretation appropriate?

Yes

Are all the source data underlying the results available to ensure full reproducibility?

Yes

Is the study design appropriate and is the work technically sound?

Yes

Are the conclusions drawn adequately supported by the results?

Partly

Are sufficient details of methods and analysis provided to allow replication by others?

Yes

Reviewer Expertise:

Mathematics, Mathematical Statistics, Data Analysis

I confirm that I have read this submission and believe that I have an appropriate level of expertise to confirm that it is of an acceptable scientific standard.

## References

[1] : An Extension of Some Truncated Distributions Family With Application. *Iraqi Journal of Science* .2025; 10.24996/ijs.2025.66.1.25 317-328 10.24996/ijs.2025.66.1.25

# Reviewer report: Reviewer response for version 1

**DOI:** 10.5256/f1000research.191964.r485142

**Published:** 2026-05-21

**Authors:** Mundher A Khaleel

**Affiliations:** 1 Department of Mathematics, College of Computer Science and Mathematics, Tikrit University, Tikrit, Iraq

This paper presents an interesting and valuable contribution to the field of bounded lifetime distributions through the development of the modified [0,1]-Right Truncated Exponential Rayleigh Distribution. The manuscript demonstrates strong theoretical effort by deriving important statistical properties such as moments, entropy, quantile function, and order statistics, in addition to applying MLE and simulation analysis. The inclusion of practical applications and model comparison using information criteria strengthens the study’s applied significance. Moreover, the graphical analysis and flexibility of the proposed model indicate its potential usefulness in reliability and survival data analysis. Overall, the study has promising scientific value and can become a strong contribution after minor improvements in presentation and mathematical clarification.

Is the work clearly and accurately presented and does it cite the current literature?

Yes

If applicable, is the statistical analysis and its interpretation appropriate?

Yes

Are all the source data underlying the results available to ensure full reproducibility?

Yes

Is the study design appropriate and is the work technically sound?

Yes

Are the conclusions drawn adequately supported by the results?

Yes

Are sufficient details of methods and analysis provided to allow replication by others?

Yes

Reviewer Expertise:

Mathematical Statistics

I confirm that I have read this submission and believe that I have an appropriate level of expertise to confirm that it is of an acceptable scientific standard.

# Reviewer report: Reviewer response for version 1

**DOI:** 10.5256/f1000research.191964.r466937

**Published:** 2026-04-06

**Authors:** E.I.Abdul Sathar

**Affiliations:** 1 University of Kerala, Trivandrum, Kerala, India

**Review Report**

 The manuscript proposes a new probability distribution called the **[0,1] Right Truncated Exponential–Rayleigh Distribution ([0,1]-RTERD)** derived from the exponential–Rayleigh distribution by applying right truncation on the interval ([0,1]). The paper derives the cumulative distribution function (CDF), probability density function (PDF), survival and hazard functions, moments, moment generating function, quantile function, skewness, kurtosis, and order statistics. The parameters are estimated using maximum likelihood estimation and the model is applied to real datasets for comparison with existing models.

 The topic is relevant to statistical modeling of **bounded lifetime data and proportion-type data**. However, the manuscript requires **significant improvements in presentation, mathematical clarity, and English language** before it can be considered for Indexing.

**Major Comments**

**1. **The manuscript introduces a truncated version of the exponential–Rayleigh distribution, but the **novel contribution is not sufficiently justified**.

 The authors should clearly explain:

- Why the **interval [0,1] truncation** is necessary.
- What advantages this model provides compared with other bounded distributions such as:
  - Beta distribution
  - Kumaraswamy distribution
  - Unit-Weibull distribution
  - Unit-Rayleigh distributions.

Currently the introduction only lists many generated families without explaining the **research gap**.

**Recommendation**

 Add a paragraph explaining:

- practical situations where **[0,1] support is required**
- why existing models are insufficient
- how the proposed distribution improves modeling flexibility.

**2. **The introduction cites several families of distributions but the discussion is **very superficial**.

 Problems:

- References are listed without explanation.
- The relationship between these models and the proposed model is unclear.
- Important literature on **unit distributions and bounded distributions** is missing.

**Recommendation**

 The authors should include literature on:

- Unit distributions
- Bounded lifetime models
- Applications in reliability and proportion data.

**3. **Several derivations are **unclear or incorrectly explained**.

 Examples:

- The derivation of the moments (Equation 9) uses series expansion but the steps are not clearly justified.
- Notation is inconsistent (for example (M_r^0)).
- Some expressions contain typographical errors.

For example:

- The transformation (y=\delta x) is introduced without properly changing the integration limits.
- The summation index appears incorrectly in the denominator in some equations.

**Recommendation**

 The mathematical derivations should be rewritten with:

- clear intermediate steps
- consistent notation
- correct integral transformations.

**4. **Some formulas for skewness and kurtosis appear incorrect.

 The paper states:

 [

 S.C = \frac{M_3^0}{(M_2^0)^{3/2}}

 ]

 and

 [

 K.C = \frac{M_4^0}{(M_2^0)^2} - 3

 ]

 However, skewness should normally use **central moments**, not raw moments.

 This needs clarification.

**5. **Figures 1–4 show the CDF, PDF, survival, and hazard functions.

 Problems:

- The captions are vague (“Description of the figure…”).
- Parameter values used in the plots are not clearly reported.
- The figures do not demonstrate the flexibility of the distribution.

**Recommendation**

 Improve the figure captions and explain:

- parameter combinations
- shapes of hazard function
- modeling implications.

**6. **The abstract claims that the model is applied to real datasets and compared with other distributions, but the details are not clearly presented.

 Important missing elements:

- dataset description
- sample size
- goodness-of-fit statistics
- comparison tables (AIC, BIC, etc.)
- graphical fitting analysis.

Without these details, the **practical value of the model cannot be assessed**.

**7. **The manuscript contains **many grammatical errors and awkward sentences**.

 Examples:

- “Right – truncated [0,1] exponential Rayleigh distribution for lifetime is presented.”
- “The peaks tend towards smaller values of (x) as (δ) increases.”
- “A spatial case of eqn. (18)” (should be *special case*).

These issues affect readability.

**Recommendation**

 The manuscript requires **major English editing by a fluent speaker or professional editing service**.

**Minor Comments**

1. Symbols should be defined when first introduced.
2. Equation numbering is inconsistent.
3. Some references appear outdated.
4. Notation such as ((δ, λ)) should be consistently formatted.
5. Table captions should clearly describe the content.
6. The paper sometimes mixes **lifetime terminology with bounded data terminology**, which may confuse readers.

**Strengths of the Paper**

- The study proposes a **bounded lifetime distribution** which may be useful for modeling proportions and reliability data.
- Several statistical properties are derived.
- The paper attempts both **theoretical and applied analysis**.
- Parameter estimation using **MLE** is included.

**Recommendation**

**Decision: Major Revision Required**

 The manuscript has potential but requires **substantial revisions**, including:

1. Clear explanation of novelty.
2. Improved literature review.
3. Correct and detailed mathematical derivations.
4. Stronger data analysis and model comparison.
5. Major language editing.

After these improvements, the paper may be reconsidered for indexing.

Is the work clearly and accurately presented and does it cite the current literature?

Partly

If applicable, is the statistical analysis and its interpretation appropriate?

Partly

Are all the source data underlying the results available to ensure full reproducibility?

No

Is the study design appropriate and is the work technically sound?

Partly

Are the conclusions drawn adequately supported by the results?

Partly

Are sufficient details of methods and analysis provided to allow replication by others?

Partly

Reviewer Expertise:

Reliability Theory

I confirm that I have read this submission and believe that I have an appropriate level of expertise to confirm that it is of an acceptable scientific standard, however I have significant reservations, as outlined above.
